# Reducing cost in DNA-based data storage by sequence analysis-aided soft information decoding of variable-length reads

**DOI:** 10.1093/bioinformatics/btad548

**Published:** 2023-09-05

**Authors:** Seong-Joon Park, Sunghwan Kim, Jaeho Jeong, Albert No, Jong-Seon No, Hosung Park

**Affiliations:** Department of Electrical and Computer Engineering, Seoul National University, Seoul 08826, South Korea; Department of Electrical, Electronic and Computer Engineering, University of Ulsan, Ulsan 44610, South Korea; Department of Electrical and Computer Engineering, Seoul National University, Seoul 08826, South Korea; Department of Electronic and Electrical Engineering, Hongik University, Seoul 04066, South Korea; Department of Electrical and Computer Engineering, Seoul National University, Seoul 08826, South Korea; Department of Computer Engineering, Chonnam National University, Gwangju 61186, South Korea; Department of ICT Convergence System Engineering, Chonnam National University, Gwangju 61186, South Korea

## Abstract

**Motivation:**

DNA-based data storage is one of the most attractive research areas for future archival storage. However, it faces the problems of high writing and reading costs for practical use. There have been many efforts to resolve this problem, but existing schemes are not fully suitable for DNA-based data storage, and more cost reduction is needed.

**Results:**

We propose whole encoding and decoding procedures for DNA storage. The encoding procedure consists of a carefully designed single low-density parity-check code as an inter-oligo code, which corrects errors and dropouts efficiently. We apply new clustering and alignment methods that operate on variable-length reads to aid the decoding performance. We use edit distance and quality scores during the sequence analysis-aided decoding procedure, which can discard abnormal reads and utilize high-quality soft information. We store 548.83 KB of an image file in DNA oligos and achieve a writing cost reduction of 7.46% and a significant reading cost reduction of 26.57% and 19.41% compared with the two previous works.

**Availability and implementation:**

Data and codes for all the algorithms proposed in this study are available at: https://github.com/sjpark0905/DNA-LDPC-codes.

## 1 Introduction

Due to the exponentially increasing digital data, new archival storage that can store a massive amount of data has been researched. DNA is known as one of the most promising mediums for a new storage system (Church *et al.* 2012, Goldman *et al.* 2013, Grass *et al.* 2015, Blawat *et al.* 2016, Bornholt *et al.* 2016, Erlich and Zielinski 2017, Yazdi *et al.* 2017, Organick *et al.* 2018). We call this DNA-based data storage. DNA-based data storage is very dense, can store over 60 petabytes per cubic centimeter, can easily copy the data, and has a high durability with low energy cost (Saiki *et al.* 1985, Nguyen *et al.* 2021, Organick *et al.* 2021). Since these characteristics of DNA-based data storage are primary factors for a future archival storage system, it is attracting many researchers as an alternative to current storage systems (Choi *et al.* 2019, 2020, Jeong *et al.* 2021).

We map binary data to four bases when storing digital data to DNA bases. Then, we write (i.e. synthesize) the sequences into the form of actual DNA molecules and obtain the DNA oligo pool. To retrieve the original data from the DNA oligo pool, we read (i.e. sequence) the DNA sequences by a sequencer and convert DNA sequences back to the original digital data. However, DNA synthesis and sequencing steps are prone to errors, and several experiments of DNA-based data storage show that the base error rate is approximately 1% when using Illumina sequencers ( Schirmer *et al.* 2016, Ceze *et al.* 2019). One of the key solutions to cope with errors is to employ error-correcting codes (ECCs) and correct these errors.

The most important and active area of research in DNA-based data storage is to reduce the cost when storing and restoring the data (Yim *et al.* 2014, Erlich and Zielinski 2017, Yazdi *et al.* 2017, Chandak *et al.* 2019, Choi *et al.* 2019, Jeong *et al.* 2021). There are mainly two different types of costs: Writing and reading costs. It would be desirable to reduce both writing and reading costs; however, there is a trade-off between writing cost (the number of synthesized bases divided by the number of information bits) and reading cost (the number of sequenced bases divided by the number of information bits) (Chandak *et al.* 2019, Heckel *et al.* 2019, Jeong *et al.* 2021). Increasing the density (bits/nt) during the encoding process (low writing cost) usually leads to an increased number of used reads during the decoding process (high reading cost), and decreasing the density (high writing cost) usually leads to a decreased number of used reads (low reading cost). Employing ECCs with high error-correction capabilities can reduce the reading cost but requires more redundancy which leads to more writing cost in DNA-based data storage. Thus, it is necessary to improve this trade-off between the two costs.

To reduce writing and reading costs, recent works adopted the encoding schemes of DNA Fountain (Erlich and Zielinski 2017), which employed Reed–Solomon (RS) codes and fountain codes [especially Luby transform (LT) codes] (Anavy *et al.* 2019, Koch *et al.* 2020, Jeong *et al.* 2021, Cao *et al.* 2022, Song *et al.* 2022, Wang *et al.* 2022). This coding scheme is an effective method to correct errors and recover lost strands (i.e. dropout), but a more robust coding scheme is still necessary for further cost reduction. Low-density parity-check (LDPC) codes have been emerging as another solution to reduce the costs in DNA-based data storage. LDPC codes are capacity-approaching ECCs that nearly achieve the Shannon limit with low complexity (Richardson and Urbanke 2008). Owing to their powerful performance, LDPC codes have been adopted for many communications systems, such as 5G new radio (NR) and digital TV broadcasting. Several works have used LDPC codes in DNA-based data storage (Yim *et al.* 2014, Chandak *et al.* 2019). For example, Chandak *et al.* (2019) applied the LDPC code to the block of binary data and then partitioned a large block into DNA oligos. The authors of Chandak *et al.* (2019) also considered synchronization markers to handle incorrect lengths of the consensus sequence. The systems in Chandak *et al.* (2019) required additional redundancy and could not efficiently handle variable-length (VL) reads and dropouts during the whole DNA-based data storage procedure. Also, when partitioning a single sequence into segments after the LDPC encoding, insertion, deletion, or erasure errors were regarded as burst errors which caused challenging problems for correction. Thus, more effective encoding and decoding algorithms should be suggested.

In this article, we propose new encoding and decoding schemes that fully use the sequenced data and reduce both writing and reading costs. Unlike previous schemes that concatenate two different ECCs or introduce additional redundancy, our encoding scheme consists of a single LDPC code to handle errors and erasure sequences (i.e. dropouts). Additionally, we embed the proposed LDPC codes to the bases in the same position of oligo sequences as an inter-oligo code. Then, all errors become a single erasure or substitution error for each LDPC codeword, which can be easily corrected. Our simple encoding algorithm achieves a low writing cost, high error-correction performance, and low computational complexity.

**Figure 1.**
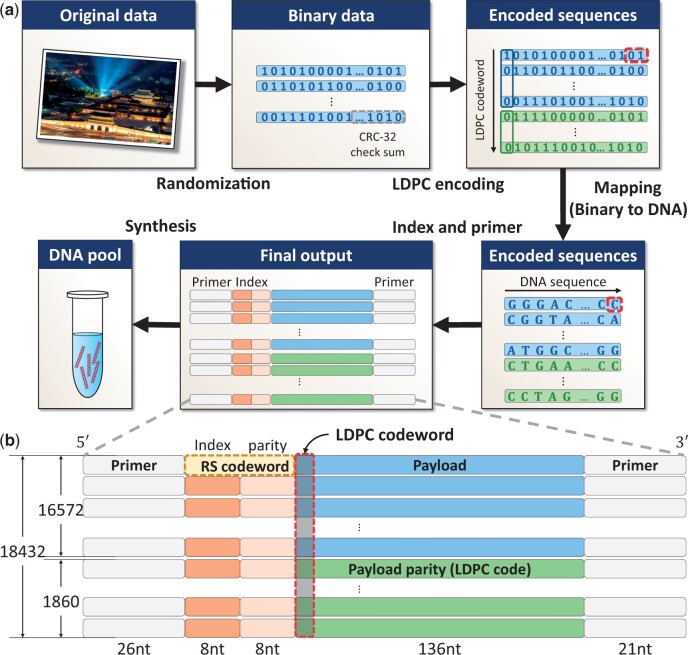
(a) Overall encoding procedure of the proposed sequence and the encoding output. The original data, the image of 584.83 KB of Gyeongbokgung Palace in Seoul, Republic of Korea, from https://en.wikipedia.org/wiki/Gyeongbokgung, are first converted to a binary sequence and partitioned into short sequences. Then, LDPC codes are employed in inter-oligo direction to generate parity sequences and convert each binary sequence to DNA sequences. For each oligo sequence, the protected index is added. Finally, primers are added and the DNA oligo sequences are synthesized. (b) Oligo data structure after the encoding. In total, 16 572 oligo sequences are from the data payload and 1860 oligo sequences are generated by LDPC encoding. Each sequence consists of 26 nt primer, 8 nt index, 8 nt RS parities for the index, 136 nt data payload or LDPC parity, and 21 nt primer. Finally, 18 432 oligo sequences with length 199 nt are generated after the encoding procedure.

In the decoding process, previous works based on the Illumina sequencer (Erlich and Zielinski 2017, Chandak *et al.* 2019, Jeong *et al.* 2021) consider the reads with the correct length (152 nt) only and discard all reads with VLs. They use the reads with only correct length because a single unreliable shortened read can cause catastrophic effects during decoding. However, there is a limitation in reducing the reading cost when reads with the correct length are used during the decoding procedure. Thus, we design a new sequence analysis-aided decoder that uses reads with correct lengths (152 nt) and VLs (150 and 151 nt). We extract reliable information from VL reads and discard erroneous reads during the proposed clustering and multiple sequence alignment (MSA) steps in the sequence analysis-aided decoder. The clustering and the MSA steps are designed to be suitable for DNA-based data storage by using edit distance (ED) and quality scores (*Q*-scores). Finally, the decoder applies an iterative LDPC re-decoding algorithm to utilize soft information of consensus reads maximally, which highly reduces the reading cost. An entire encoding and decoding process is depicted in Fig. 1(a).

This work achieves a writing cost of 0.62 bases/bit and a reading cost of 2.41 bases/bit on average. To show the reading cost reduction of the proposed scheme, we conduct a practical DNA-based data storage experiment. We synthesize 548.83 KB of an image file in the form of DNA and perfectly retrieve the original data while reducing both writing and reading costs compared with the two previous works Erlich and Zielinski (2017) and Jeong *et al.* (2021). This work achieves the writing cost of 0.62 bases/bit, which is 7.46% reduced compared with Erlich and Zielinski (2017) and Jeong *et al.* (2021), respectively. Also, the average reading cost of experiments is 2.41 bases/bit, which is 26.57% and 19.41% reduced for the same sequencing environment compared with Erlich and Zielinski (2017) and Jeong *et al.* (2021), respectively. As a result, we demonstrate that our work reduces 7.46% of a writing cost and 26.57%, 19.41% of a reading cost on average compared with the results in Erlich and Zielinski (2017) and Jeong *et al.* (2021), respectively. Both the encoding and decoding algorithms can be applied to any other digital data that need to be stored without any restrictions.

## 2 Materials and methods

### 2.1 Encoding procedure

Figure 1(a) shows the overall encoding procedure of the proposed scheme. The encoding procedure follows similar steps with Blawat *et al* (2016), whose work are well-known for their efficiency in DNA-based data storage (Blawat *et al.* 2016, Chandak *et al.* 2019). It consists of a strongly protected index and an inter-oligo code. Unlike previous schemes that concatenate two different codes or introduce additional redundancy [e.g. intra-oligo code (Blawat *et al.*, 2016) or synchronization markers (Chandak *et al.*, 2019)], our encoding scheme handles both errors and erasure sequences by employing a single LDPC code.

We first convert a 548.83-KB image file to a long binary sequence. Then, we randomize the binary sequence by XOR with a pseudo-random binary sequence to prevent long homopolymer runs and high GC-content since it is known that DNA sequences without constraints are prone to errors (Schwartz *et al.* 2012, Ross *et al.* 2013). Although homopolymer run and GC-content constraints are not satisfied perfectly, this process still gives a good quality during the synthesis. We attach 32 bits of cyclic redundancy check-32 (CRC-32) checksum in the suffix of binary sequence for error detection after the decoding. The binary sequence is partitioned into non-overlapping 16 572 fragments of sequences of length 272 bits.

Then, we employ LDPC codes as ECCs in an inter-oligo direction. From several candidates of LDPC codes, we choose RS code-based LDPC (RS-LDPC) codes, which is a regular LDPC with high performance (Djurdjevic *et al.* 2003). The key point in the encoding procedure is that we use only inter-oligo codes, unlike previous schemes that considered both inter-oligo and intra-oligo codes. When LDPC codes are applied through all DNA sequences, insertion, deletion, or erasure errors are treated as a single erasure or substitution error in the viewpoint of each codeword in the inter-oligo direction. In other words, no additional intra-oligo code or redundancy is required to handle all errors in DNA-based data storage in the proposed scheme. Also, since we use soft decision decoding of LDPC codes, several techniques, such as VL reads and *Q*-score, can be applied during the decoding procedure. We apply LDPC encoding for every 272 binary positions of 16 572 and add 1860 LDPC redundancy binary sequences in the inter-oligo direction. After mapping two bits to one base (i.e. *A *=* *00, *C *=* *01, *G *=* *10, *T *=* *11), we have 18 432 oligo sequences of length 136 nt.

The next step is to attach an index to each oligo sequence. These indices should be protected with robust codes to perform index-based clustering. To order 18 432 oligo sequences, we need at least $\left\lceil {\;\text{log}\;}_{2}18,432\right\rceil =15$ bits, and thus we use an 8-nt long index. Rather than choosing indices in ascending order (from AAAAAAAA to GATTTTTT), we choose the best 18 432 indices that are robust to errors. From 4^8^ index candidates, we eliminate sequences with homopolymer runs exceeding 3 and obtain 18 432 different indices. Then, we protect the index with a robust RS code since it would be catastrophic when errors in the index are not corrected properly. We add 8 nt RS redundancy to the 8 nt index by using (8,4) RS code over GF(2^4^), where each symbol is 2 nt. The minimum Hamming distance (HD) is 5, which can correct up to two symbols and detect up to four symbols. As a result, we get 18 432 oligo sequences of length 152 nt in total. Finally, we attach the sequence primers in both prefixes and suffixes of all oligo sequences and synthesize the final output of length 199 nt to a DNA oligo pool. Figure 1(b) is the final output after encoding the original data.

### 2.2 Decoding procedure

To retrieve the original data from DNA sequences, we first read the DNA oligo pool by Illumina MiSeq. Then, we need some preprocessing steps for the decoding procedure to restore the data perfectly. From reads sequenced by Illumina sequencer, we randomly sample the forward and reverse reads and assemble the reads using paired-read merger FLASH (Magoč and Salzberg 2011). One of the key ideas to aid the decoding performance is that this work uses extra reads (shortened reads of 150 and 151 nt long) rather than using reads with only 152 nt from merged reads. Since the percentage of shortened reads is higher than that of lengthened reads and through several trials of decoding experiments, using additional reads with deletion errors improves the performance. We extract as much information as possible from the shortened reads and use this additional information during the decoding process.

#### 2.2.1 Clustering and MSA

In DNA-based data storage, clustering and alignment steps are essential to retrieve the original data efficiently. One of the most recent works (Jeong *et al.* 2021) applied HD-based clustering and improved the decoding performance. However, the decoder in Jeong *et al.* (2021) can handle the reads with only the correct length and discard all reads with incorrect lengths. Here, we propose clustering and MSA steps that can handle VL reads efficiently, which can discard abnormal reads and extract only high-quality ones for the decoder.

First, in the clustering step, we check the first 16 nt (which should be 8 nt index + 8 nt RS parity) from chosen reads and figure out the original index by the RS decoding. Since the indices are protected with strong RS codes, most are corrected during the RS decoding step in this work. Based on the RS decoding result, the reads with the same decoded index are gathered together into the same cluster. Since we make clusters according to their index values, we call this process an *index-based clustering*.

One problem that can occur after the RS decoding is that some indices can be decoded into the wrong indices. Even if a single decoded index is clustered with the wrong index, it will cause a catastrophic effect during the next steps of the decoding procedure. To prevent this problem, we find these abnormal reads by calculating the EDs of each read with all other reads in the same cluster. If one read has the ED larger than a threshold *d_th_*, we consider it an abnormal read and discard it from the cluster. We call this process an *ED-based clustering*. By several trials, we choose *d_th_* = 10 in our experiments. By combining two clustering methods, we can group reads properly.

After the clustering step, we align the VL reads in each cluster with the MSA algorithm. To properly align reads with different lengths, we need to consider several conditions when processing the MSA algorithm. For each cluster, we first check which cluster satisfies the following conditions.

1. All reads have the correct length (i.e. 152 nt).
2. Not all reads have the correct length.

If the cluster satisfies condition 1, reads in the cluster do not proceed MSA algorithm. They pass all the following conditions and are directly used in the log-likelihood ratio (LLR) calculation step. If the cluster satisfies condition 2, we consider the following conditions.

1. The size of the cluster is 1.
2. The size of the cluster is more than 1.

Condition 3 is when a single shortened read exists in the cluster. When a deletion error occurs in a DNA sequence, we do not know which base in the sequence is deleted, and all the subsequent bases become shifted ahead by one position. Thus, the bases after the deletion are regarded as having a high probability of substitution error when we estimate the true sequence relying on the absolute positions of bases. However, we know the first or the last base is always correct when we consider the relative position unless an error occurs in the position. Also, it is known that asymmetric errors occur in DNA-based data storage, and the last base of the oligo sequence is more prone to errors compared with other positions (Organick *et al.* 2018). Thus, the decoding performance of the last two codewords (last bases) is the bottleneck of the decoding performance of DNA-based data storage, and we use the last base of this sequence if it seems reliable rather than discarding this whole sequence like before.

To determine whether the last base is reliable, we check the *Q*-score value. Usually, bases with low *Q*-score tend to have more base errors (Li *et al.* 2008, Jeong *et al.* 2021). To use only highly reliable bases, we save the last base only if the *Q*-score of the last base is higher than 30 and directly use them for LLR calculation. For other bases, we consider them as erasure bases when calculating LLR. Although we store only the last base, this process improves the decoding performance of DNA-based data storage. For condition 4, we align the reads using the MSA algorithm. Here, we use the program called MUSCLE (Edgar 2004). For these clusters, we go to the final step.

Since the length of the output reads can be different from the input reads after the MSA step, we again check the lengths of the output reads.

1. The output reads have the correct length (i.e. 152 nt).
2. The output reads do not have the correct length (i.e. 152 nt).

If the cluster satisfies condition 5, we use all reads to calculate LLR values. If the cluster satisfies condition 6, we can say that the alignment is not done correctly, and several insertion and deletion errors occur. This case usually happens when the cluster size is 2 and two reads are from totally different sequences, but decoded indices are the same. In this case, as we have done in condition 3, we save the last base if the Q-score is higher than 30 and use them for LLR calculation rather than discarding a whole sequence.

#### 2.2.2 LLR calculation using *Q*-score

The last preprocessing step is LLR calculation for soft decision decoding. For LLR calculation, DNA sequences in each cluster should be converted to binary sequences and soft LLR values are computed for all clusters based on the equation in the work (Chandak *et al.* 2019). In Chandak *et al.* (2019), authors count the numbers of 0’s and 1’s for a bit position of reads in the same cluster and the reliability of LLR values depends on the number of the same counts. Rather than using the same LLR calculation method, we modified it according to our scheme, shown in the next subsection.

To maximize the quality of LLR values, we only count the numbers 0 and 1 if the *Q*-score of the last base is higher than 20 when computing LLR in the last two bits. As mentioned above, low *Q*-scores tend to have more base, and consensus errors, and the last base is more prone to errors. Thus, maximizing the decoding performance of the last two codewords improves the decoding performance of the DNA-based data storage. By discarding unreliable bits, we obtain the decoding performance gain compared with the original LLR calculation, shown in the next section.

For the indices where no reads are detected (i.e. clusters of size 0), we consider them as erasures and set the LLR value to zeros for all positions. The erased bits from these indices can be corrected by using LDPC codes. Since the LDPC codes are employed in the orthogonal direction to erasure sequences, a sequence erasure is considered a single erasure for each LDPC code. When LLR values for all 18 432 oligo sequences are computed, we align them in an index order and start decoding LDPC codes for the inter-oligo direction. The important point is that all these techniques using *Q*-score and handling erasure oligos are available since we use soft decision decoding of LDPC codes.

#### 2.2.3 Iterative re-decoding of LDPC codes

In this work, we propose an iterative re-decoding stage of LDPC codes using the characteristic of LDPC codes. One of the characteristics of LDPC codes is that by using the parity-check matrix of LDPC codes, we can calculate the syndrome vector and determine whether the decoded codeword is correct. In this step, we first perform a conventional decoding of LDPC codes using the initial LLR values. If the decoding fails (even if a single codeword fails), the re-decoding stage is activated only for codewords that failed in decoding. We recompute the input LLR values when the first decoding fails by subtracting Δϵ from *ϵ*. The decoder does this work iteratively until all codewords succeed in decoding or when *ϵ* reaches a predetermined minimum value ($\epsilon_{\text{end}}$). This iterative re-decoding stage requires just a low additional complexity since the re-decoding is usually performed over only a few codewords. The modified LLR calculation considering the re-decoding step is as follows:

(1) $$\begin{array}{c} {\text{LLR}}^{i}(k_{0},k_{1},k_{e})=\ln\frac{P^{i}\left((k_{0},k_{1},k_{e})|0\right)}{P^{i}\left((k_{0},k_{1},k_{e})|1\right)}\\ =(k_{0}-k_{1})\ln\frac{1-(\epsilon -i\Delta \epsilon )}{\left(\epsilon -i\Delta \epsilon \right)}, \end{array}$$

where *i* denotes the iteration number of the re-decoding step and *k*_0_, *k*_1_, and *k_e_* denote the numbers of 0’s, 1’s, and erasure for a bit position of reads in the same cluster, respectively. Since erased bits do not give any information, we do not use them in the LLR calculation. By several trials, Δϵ is determined to be 0.005 and the initial value of *ϵ* to be 0.02. When all LDPC codewords are corrected, we check whether the retrieved data is perfect by the CRC-32 decoder.

### 2.3 DNA synthesis and sequencing

We store a 548.83-KB image file, resulting in 18 432 oligo sequences of length 152 nt synthesized into a DNA oligo pool with sequence primers (5′-side: GTTCAGAGTTCTACAGTCCGACGATC and 3′-side: TGGAATTCTCGGGTGCCAAGG) added at both ends of 152 nt long oligo sequence. The DNA oligo pool is synthesized by Twist Bioscience.

We perform DNA sequencing three times for the same DNA oligo pool for more accurate and reliable results. To have the same sequencing environment with previous works (Erlich and Zielinski 2017, Jeong *et al.* 2021), we follow the same sequencing procedure with these works. We use Q5 Hot Start High-Fidelity 2X Master Mix with Illumina small RNA primers RP1 and RPI1 for PCR [2.5 µl of each primer (10 µM), 25 µl Q5 Master Mix in a 50-µl reaction]. Thermocycling conditions for PCR: 97°C for 30 s, 10 cycles of 98°C for 10 s, 60°C for 30 s, 72°C for 30 s, 5 min of extension at 72°C. The library is purified 1:1, cleaned up with Agencourt AMPure XP, and eluted in 20 µl water. The sequencing is performed by Cosmogenetech using the Illumina Miseq Reagent v3 kit (600 cycles) with 150 pair-end reads in both forward and reverse directions. For three sequencing experiments, Q30 is 96.23%, 96.28%, and 95.71%, respectively.

## 3 Results

### 3.1 Experimental results

We choose the image file of size 548.83 KB (16 572 × 136 nt) and encode it to the output data size of 18 432 × 152 nt. Figure 1(b) shows that the output data size of 18 432 × 152 nt with 26 nt sequence primers in the prefix and 21 nt sequence primers in the suffix. The information density of the proposed method is 1.61 bits/nt, whose writing cost is 0.62 bases/bit. In Jeong *et al.* (2021), the authors encoded the data by using the coding scheme of DNA Fountain. They encoded the data of size 513.6 KB to 18 000 × 152 nt, which has an information density of 1.50 bits/nt and a writing cost of 0.67 bases/bit. In terms of the writing cost, the proposed encoding scheme reduced the writing cost by 7.46% compared with the work (Jeong *et al.* 2021). Then, in Jeong *et al.* (2021), the authors measure the decoding performance of two decoders, which are Erlich’s decoder proposed in Erlich and Zielinski (2017) and Jeong’s decoder proposed in Jeong *et al.* (2021).

After we obtain the reads after the sequencing, we first merge forward and reverse reads using FLASH. The percentages of merged reads are over 95% on average, and from merged reads, 93.01%, 93.36%, and 92.77% are the reads with length 152 nt. Since the proposed decoder uses additional reads of length 150 and 151 nt for input, we use extra 1.31%, 1.33%, and 1.45% of shortened reads for Exp. #1, #2, and #3, respectively. Also, by applying the sequenced analysis-based decoder, 6.09 abnormal reads are discarded during the ED-based clustering. This result shows that the ED-based clustering method protected 6.09 clusters out of 18 432 clusters from being ruined. In addition, we use extra 29.7 and 17.14 reads from reads that satisfy conditions 3 and 6 by using *Q*-score information during the MSA step.

In the experiment, we use the same synthesis provider (Twist Bioscience) and the sequencing kit (Illumina Miseq v3 kit) in the same sequencing environment and measure the decoding performance of the proposed decoder. Table 1 shows the number of successful decoding trials out of 200 trials, according to the random sampling numbers using the proposed decoder, Erlich’s decoder, and Jeong’s decoder. It is worth noting that Table 1 shows the best results of Erlich’s and Jeong’s decoders from their multiple trials. For the three experiments, Exp #1, #2, and #3, the minimum random sampling numbers are 72 500, 71 500, and 73 500, and the reading costs are 2.44, 2.41, and 2.48 bases/bit for Exp #1, #2, and #3, respectively. Thus, we can recover 548.83 KB of data stored in the DNA with a median coverage of only 3.93 reads per DNA sequence. Erlich’s and Jeong’s minimum random sampling numbers are 90 000 and 82 000, and the reading costs are 3.33 and 3.03 bases/bit, respectively. These are the best results among their experiments. On average, the decoder of this work reduces the reading costs by 26.57% and 19.41% compared with the two referenced works. In conclusion, this work achieves a high reduction of writing cost by 7.46% and reading cost by 26.57% and 19.41% in DNA-based data storage, compared with the works using decoders of Erlich’s and Jeong’s, respectively.

**Table 1.** The decoding success number using the proposed decoder for Exp #1, #2, #3, Erlich’s decoder proposed in Erlich and Zielinski (2017), and Jeong’s decoder proposed in Jeong *et al.* (2021) out of 200 trials according to the random sampling number

	This work				
Random sampling	Exp #1	Exp #2	Exp #3	Erlich’s	Jeong’s
69 000		168			
69 500	129	193			
70 000	147	193			
70 500	182	199	151		
71 000	184	199	178		
71 500	194	**200**	192		
72 000	199		197		
72 500	**200**		197		
73 000			199		
73 500			**200**		
76 000				6	138
78 000				56	173
80 000				143	193
82 000				179	**200**
84 000				187	
86 000				196	
88 000				198	
90 000				**200**	

*Notes*: All experiments have the same sequencing environment and the same sequencer. For all three experiments, the minimum random sampling number where all 200 trials of decoding succeed are 72 500, 71 500, and 73 500, respectively. Boldface letters represent the first moment when all the trials succeed.

**Figure 2.**
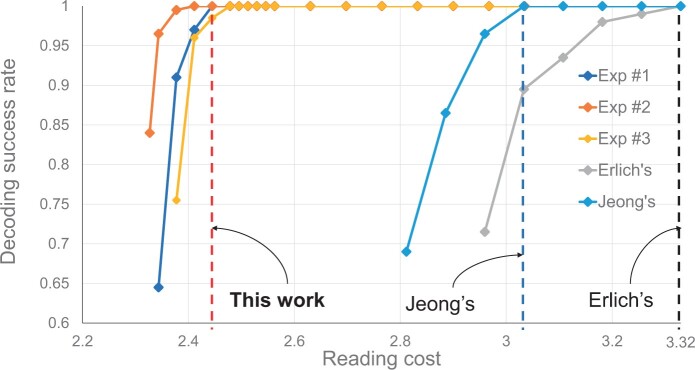
The graph of decoding success rate versus reading cost, using the proposed decoder for the three experiments, Erlich’s decoder (Erlich and Zielinski 2017) and Jeong’s decoder (Jeong *et al.* 2021). The results of Erlich’s and Jeong’s are their best results. The red dashed line is the average minimum reading cost (2.44) of Exp #1, #2, and #3 when the decoding success rate is 1. The blue and black dashed lines are the minimum reading costs of Jeong’s decoder (3.03) and Erlich’s decoder (3.33), respectively. Each sample point represents a random sampling number shown in Table 1.

Figure 2 shows the plots of Table 1 for three different decoders. Each sample point represents the corresponding random sampling numbers in Table 1. We see that the decoding success rate falls rapidly when the reading cost becomes lower, and the proposed decoder highly reduces the reading cost compared with the two decoders of Erlich and Zielinski (2017) and Jeong *et al.* (2021).

### 3.2 Effects of using VL reads and *Q*-score

To demonstrate the effects of using VL reads and *Q*-score during clustering, MSA, and LLR calculation steps, we conduct another simulation with the decoder using only 152 nt long reads without *Q*-score. From now on, we call this decoder Decoder A. Since the decoding result of Exp #1 is the average performance of three experiments, these results are the average value of 200 trials of Exp #1 at the minimum random sampling number 72 500.

Table 2 is the comparison between the Decoder A and the proposed decoder. The number of used reads for Decoder A is 67 452.97, which is 93.01% of the randomly sampled reads for decoding. Since the proposed decoder uses additional shortened reads for the decoding, the proposed decoder uses 94.41% of the randomly sampled reads, which is 68 387.09. Also, due to applying strong ECC in the index, the proposed decoder corrected the index of 1570.41 reads by RS codes, which is 64.14 more reads compared with Decoder A. From 18 432 DNA sequences, Decoder A has 909.04 erasure sequences. Compared with Decoder A, the proposed decoder has 877.70 erasures, which is 31.34 less than Decoder A. Finally, for the random sampling number of 72 500, the proposed decoder succeeds in all 200 trials of decoding, but Decoder A succeeds in 179 trials, which is 21 less than the proposed decoder. This difference comes from the effect of using VL reads and *Q*-score during the decoding procedure.

**Table 2.** Detailed comparison between Decoder A and the proposed decoder

Decoders (Exp. #1)	**Decoder A**	This work	
Total used reads	67 432.90	68 387.09	(+954.19)
Used reads/total reads	93.01%	94.41%	(+1.36)
Corrected index	1506.27	1570.41	(+64.14)
Erasure sequences	909.04	877.70	(–31.34)
(cluster size 0)			
Decoding performance	179	200	(+21)

*Note*: The comparison between the decoder using only 152 nt long reads without *Q*-score (Decoder A) and the decoder using reads with 150, 151, and 152 nt long with *Q*-score (proposed decoder).

### 3.3 Comparison

Table 3 shows the comparison between this work and previous works using Erlich’s decoder and Jeong’s decoder. In work Jeong *et al.* (2021), the authors encoded the data of size 513.6 KB into DNA sequences, which has an information density of 1.50 bits/nt and the writing cost of 0.6659 bases/bit using the encoding method proposed in Erlich and Zielinski (2017). Then, in Jeong *et al.* (2021), they measure the decoding performance by applying two different decoders [Erlich and Zielinski’s (2017) and Jeong *et al.*’s (2021)]. In this work, we store data of size 548.8 KB into DNA sequences, corresponding to the information density of 1.61 bits/nt and the writing cost of 0.62 bases/bit. In terms of the writing cost, the proposed encoding scheme reduced the writing cost by 7.46% compared with the two referenced works. Unlike the two referenced works, which employ LT codes as the inter-oligo code and RS codes as the intra-oligo code, this work employs LDPC codes for the inter-oligo code and no other code is employed in the intra-oligo direction.

**Table 3.** Detailed comparison between this work and the two previous work using Erlich’s and Jeong’s decoder

Method	This work	Erlich’s Erlich and Zielinski (2017)	Jeong’s Jeong *et al.* (2021)
Data size	548.8 KB	513.6 KB	513.6 KB
Index protected?	Yes (RS codes)	No	No
Information density	1.61 bits/nt	1.50 bits/nt	1.50 bits/nt
Writing cost	0.62 bases/bit	0.67 bases/bit	0.67 bases/bit
Inter-oligo code	LDPC code	LT code	LT code
Intra-oligo code	–	RS code	RS code
Length of used reads	150, 151, 152 nt	152 nt	152 nt
Clustering method	Index-based, ED-based	Sequence-based	HD-based
Use *Q*-score?	Yes	No	Yes
Average coverage	3.93	5	4.56
Reading cost	2.44 bases/bit	3.33 bases/bit	3.03 bases/bit

*Notes*: All three works store digital files in a DNA oligo pool. All three works synthesized DNA oligo pool in Twist Bioscience and use Illumina Miseq v3 kit in the same sequencing environment. They all employed ECCs, but this work employed an inter-oligo code only. As you can see, this work improves both writing and reading costs compared with the two referenced works.

For the decoding, only the proposed scheme uses VL reads of 150, 151, and 152 nt and the other two works use only the reads with length 152 nt. For the clustering, Erlich’s decoder used only sequence-based clustering, which only gathers the same reads. Similar to Erlich’s decoder, Jeong’s decoder also used sequence-based clustering but applied HD-based clustering to discard abnormal reads. However, this work uses index-based clustering that can be applied since indices are protected during the encoding procedure. Moreover, we applied an ED-based clustering step to discard abnormal reads in each cluster if they have a large ED compared with other reads. Unlike HD-based clustering, which considers only substitution errors, ED-based clustering can be effective against insertion and deletion errors that often occur in DNA-based data storage.

This work and Jeong’s decoder use a *Q*-score during the decoding procedure. Jeong’s decoder uses a *Q*-score only to sort cluster size 1 reads. However, our work uses it to extract reliable information after the clustering and MSA steps and use this information for the soft decision decoding of LDPC codes. In terms of decoding performance, the proposed scheme successfully decodes for all 200 trials at the average random sampling number of 72 500. Erlich’s and Jeong’s decoders succeed in decoding for all 200 trials at the random sampling numbers 90 000 and 82 000. Thus, the coverages of the three works are 3.93, 5, and 4.56, respectively. As a result, our work achieves a high cost reduction on average compared with costs using Erlich’s and Jeong’s decoders, respectively.

## 4 Discussion and conclusion

Here, we proposed an effective encoding algorithm that requires a low writing cost while achieving a high reduction in the reading cost. We adopted a single LDPC code as an inter-oligo code, and this algorithm can handle all errors (i.e. substitution, insertion, deletion, and erasure) without any additional redundancy or algorithm (e.g. erasure codes and markers). During a general decoding procedure, there is a limitation in reducing the reading cost when the decoder uses reads with only the correct length. To cope with this problem, we proposed the sequence analysis-aided decoder that uses additional VL reads. We demonstrated that this work gives a writing cost reduction of 7.46% and a reading cost reduction of 26.57% and 19.41% compared with the two referenced works.

Our work can be applied to other types of synthesis that allow very short oligos or other types of sequencing, such as Oxford nanopore sequencing, the proposed encoding and decoding methods can still handle them. If very short oligos are synthesized for the same amount of data, longer LDPC codes are required as inter-oligo codes for the encoding scheme. It is worth noting that LDPC codes support a large range of blocklengths and code rates (i.e. 5G NR LDPC codes; Richardson and Kudekar 2018). Also, the nanopore sequencing is known to have higher substitution, insertion, and deletion error rates compared with Illumina sequencing (Yazdi *et al.* 2017, Chandak *et al.* 2020). Our work is robust to high error rate of the nanopore sequencing technique since we adopted LDPC codes, which is one of the most powerful ECCs that nearly achieve the capacity. Also, high insertion and deletion error rates of the nanopore sequencing incur more erasure sequences, which would give a fatal effect in previous works not utilizing VL reads. However, our work utilizes VL reads by the proposed clustering and MSA algorithms, which would make our work more robust in the nanopore sequencing technique.

## Supplementary Material

btad548_Supplementary_Data

## References

[btad548-B1] Anavy L , Vaknin I, Atar O et al Data storage in DNA with fewer synthesis cycles using composite DNA letters. Nat Biotechnol 2019;37:1229–36. 31501560 10.1038/s41587-019-0240-x

[btad548-B2] Blawat M , Gaedke K, Hütter I et al Forward error correction for DNA data storage. Procedia Comput. Sci 2016;80:1011–22.

[btad548-B3] Bornholt J , Lopez R, Carmean DM et al A DNA-based archival storage system. ACM 2016;9:6582.

[btad548-B4] Cao B , Zhang X, Cuil S et al Adaptive coding for DNA storage with high storage density and low coverage. NPJ Syst Biol Appl 2022;8:23. 35788589 10.1038/s41540-022-00233-w PMC9253015

[btad548-B5] Ceze L , Nivala J, Strauss K. Molecular digital data storage using DNA. Nat Rev Genet 2019;20:456–66. 31068682 10.1038/s41576-019-0125-3

[btad548-B6] Chandak S et al Improved read/write cost tradeoff in DNA-based data storage using LDPC codes. In: *2019 57th Annual Allerton Conference on Communication, Control, and Computing (Allerton)*. IEEE, 2019, 147–56.

[btad548-B7] Chandak S et al Overcoming high nanopore basecaller error rates for DNA storage via basecaller-decoder integration and convolutional codes. In: *ICASSP 2020–2020 IEEE International Conference on Acoustics, Speech and Signal Processing (ICASSP)*. IEEE, 2020, 8822–6.

[btad548-B8] Choi Y , Bae HJ, Lee AC et al DNA micro-disks for the management of DNA-based data storage with index and write-once-read-many (WORM) memory features. Adv Mater 2020;32:2001249. 10.1002/adma.202001249 32725925

[btad548-B9] Choi Y , Ryu T, Lee AC et al High information capacity DNA-based data storage with augmented encoding characters using degenerate bases. Sci Rep 2019;9:6582. 31036920 10.1038/s41598-019-43105-w PMC6488701

[btad548-B10] Church GM , Gao Y, Kosuri S. Next-generation digital information storage in DNA. Science 2012;337:1628. 22903519 10.1126/science.1226355

[btad548-B11] Djurdjevic I , Xu J, Abdel-Ghaffar K et al A class of low-density parity-check codes constructed based on Reed–Solomon codes with two information symbols. IEEE Commun Lett 2003;7:317–9.

[btad548-B12] Edgar RC. MUSCLE: Multiple sequence alignment with high accuracy and high throughput. *Nucleic Acids Res* 2004;32:1792–7. 10.1093/nar/gkh340 PMC390337 15034147

[btad548-B13] Erlich Y , Zielinski D. DNA fountain enables a robust and efficient storage architecture. Science 2017;355:950–4. 28254941 10.1126/science.aaj2038

[btad548-B14] Goldman N , Bertone P, Chen S et al Towards practical, high-capacity, low-maintenance information storage in synthesized DNA. Nature 2013;494:77–80. 23354052 10.1038/nature11875 PMC3672958

[btad548-B15] Grass R , Heckel R, Puddu M et al Robust chemical preservation of digital information on DNA in silica with error-correcting codes. Angew Chem Int Ed Engl 2015;54:2552–5. 25650567 10.1002/anie.201411378

[btad548-B16] Heckel R , Mikutis G, Grass RN. A characterization of the DNA data storage channel. Sci Rep 2019;9:9663. 31273225 10.1038/s41598-019-45832-6 PMC6609604

[btad548-B17] Jeong J , Park S-J, Kim J-W et al Cooperative sequence clustering and decoding for DNA storage system with fountain codes. Bioinformatics 2021;37:3136–43. 33904574 10.1093/bioinformatics/btab246

[btad548-B18] Koch J , Gantenbein S, Masania K et al A DNA-of-things storage architecture to create materials with embedded memory. Nat Biotechnol 2020;38:39–43. 31819259 10.1038/s41587-019-0356-z

[btad548-B19] Li H , Ruan J, Durbin R. Mapping short DNA sequencing reads and calling variants using mapping quality scores. Genome Res 2008;18:1851–8. 18714091 10.1101/gr.078212.108 PMC2577856

[btad548-B20] Magoč T , Salzberg SL. FLASH: fast length adjustment of short reads to improve genome assemblies. Bioinformatics 2011;27:2957–63. 21903629 10.1093/bioinformatics/btr507 PMC3198573

[btad548-B21] Nguyen BH , Takahashi CN, Gupta G et al Scaling DNA data storage with nanoscale electrode wells. Sci Adv 2021;7:eabi6714. 34818035 10.1126/sciadv.abi6714 PMC8612674

[btad548-B22] Organick L , Nguyen BH, McAmis R et al An empirical comparison of preservation methods for synthetic DNA data storage. Small Methods 2021;5:e2001094. 34928102 10.1002/smtd.202001094

[btad548-B23] Organick L , Ang SD, Chen Y-J et al Random access in large-scale DNA data storage. Nat Biotechnol 2018;36:242–8. 29457795 10.1038/nbt.4079

[btad548-B24] Richardson T , Kudekar S. Design of low-density parity check codes for 5G new radio. IEEE Commun Mag 2018;56:28–34.

[btad548-B25] Richardson TJ , Urbanke R. Modern Coding Theory. Cambridge, UK: Cambridge University Press, 2008.

[btad548-B26] Ross MG , Russ C, Costello M et al Characterizing and measuring bias in sequence data. Genome Biol 2013;14:R51. 23718773 10.1186/gb-2013-14-5-r51 PMC4053816

[btad548-B27] Saiki RK , Scharf S, Faloona F et al Enzymatic amplification of beta-globin genomic sequences and restriction site analysis for diagnosis of sickle cell anemia. Science 1985;230:1350–4. 2999980 10.1126/science.2999980

[btad548-B28] Schwartz JJ , Lee C, Shendure J. Accurate gene synthesis with tag-directed retrieval of sequence-verified DNA molecules. Nat Methods 2012;9:913–5. 22886093 10.1038/nmeth.2137 PMC3433648

[btad548-B29] Schirmer M , D’Amore R, Ijaz UZ et al Illumina error profiles: resolving fine-scale variation in metagenomic sequencing data. BMC Bioinformatics 2016;17:125. 26968756 10.1186/s12859-016-0976-y PMC4787001

[btad548-B30] Song L , Geng F, Gong Z-Y et al Robust data storage in DNA by de Brujin graph-based de novo strand assembly. Nat Commun 2022;13:5361. 36097016 10.1038/s41467-022-33046-w PMC9468002

[btad548-B31] Wang P , Mu Z, Sun L et al Hidden addressing encoding for DNA storage. Front Bioeng Biotechnol 2022;10:916615. 35928958 10.3389/fbioe.2022.916615 PMC9344065

[btad548-B32] Yazdi SHT , Gabrys R, Milenkovic O. Portable and error-free DNA-based data storage. Sci Rep 2017;7:5011. 28694453 10.1038/s41598-017-05188-1 PMC5503945

[btad548-B33] Yim AK-Y , Yu AC-S, Li J-W et al The essential component in DNA-based information storage system: robust error-tolerating module. Front Bioeng Biotechnol 2014;2:49. 25414846 10.3389/fbioe.2014.00049 PMC4222239

