# Correction to: Reducing cost in DNA-based data storage by sequence analysis-aided soft information decoding of variable-length reads

**DOI:** 10.1093/bioinformatics/btad697

**Published:** 2023-11-21

This is a correction to: Seong-Joon Park, Sunghwan Kim, Jaeho Jeong, Albert No, Jong-Seon No, Hosung Park, Reducing cost in DNA-based data storage by sequence analysis-aided soft information decoding of variable-length reads, *Bioinformatics*, Volume 39, Issue 9, September 2023, btad548, https://doi.org/10.1093/bioinformatics/btad548

In the originally published version of this manuscript, author Sunghwan Kim’s affiliation was incomplete.

This has been corrected.

