# Supplementary material for: Reducing cost in DNA-based data storage by sequence analysis-aided soft information decoding of variable-length reads
Source: Bioinformatics. 2023 Sep 5;39(9):btad548. doi: 10.1093/bioinformatics/btad548 (PMC10500082; doi:10.1093/bioinformatics/btad548)
Supplement: btad548_Supplementary_Data [file btad548_supplementary_data.pdf]

Supplementary Information for

Reducing cost in DNA-based data storage by sequence analysis-aided soft information decoding of variable-length reads

Seong-Joon Park, Sunghwan Kim, Jaeho Jeong, Albert No, Jong-Seon No, and Hosung Park

Supplementary Notes 1

Encoding algorithm

Overview of the encoding algorithm

1. Convert a given file to a binary sequence.
2. Add CRC-32 checksum.
3. Partition the binary sequence into short binary sequences.
4. Apply LDPC encoding in an inter-oligo direction.
5. Append the encoded index in the prefix of all partitioned sequences.
6. Convert the appended binary sequences to DNA oligo sequences.

Fig. S2-1 shows the overall encoding algorithm of the proposed method.

Input data and CRC-32

In this work, we encoded an image file of Gyeongbokgung Palace (Supplementary Fig. S1) of size 548.83 kB. We converted it to the binary sequence and scrambled the data to avoid too-long homopolymer runs or too-high GC-content. Then, we attached the cyclic redundancy check-32 (CRC-32) checksum at the suffix of the sequence. We use CRC-32 polynomials from “Best CRC Polynomials”, by Philip Koopman (https://users.ece.cmu.edu/~koopman/crc/index.html). Then, we partitioned it into 16,572 short binary sequences of length 272 bits and apply the encoding of Reed Solomon-based low-density parity-check (RS LDPC) codes in an inter-oligo direction.

RS LDPC codes

LDPC codes are capacity-approaching code that nearly achieves the Shannon capacity with low complexity [1]. Thus, employing LDPC codes can achieve high error correction performance even with low computational complexity compared to other error-correcting codes. RS-LDPC codes are a class of LDPC codes, which are (γ,ρ)-regular codes with $\gamma p^s \times \rho p^s$ parity check matrix over $\mathrm{GF}(2)$ defined as follows.

$$\mathrm{H}_{\mathrm{GA}}(\gamma)=\begin{bmatrix}\mathrm{A}_1\\ \vdots\\ \mathrm{A}_\gamma\end{bmatrix},$$

where $1 \le \gamma \le p^s$, $2 \le \rho \le p^s$, and A_i's are $(1,\rho)$-regular matrix with size $p^s \times \rho p^s$. In

consideration of the required number and length of the sequences, we chose $p = 2$, $s = 8$, $\rho = 72$, and $\gamma = 64$ as parameters. As mentioned in [2], the lower bound of the minimum distance is as follows:

$$\mathrm{d}_{\min}(\gamma) \geq \begin{cases} \gamma + 1, \text{for odd } \gamma, \\ \gamma + 2, \text{for even } \gamma. \end{cases}$$

Thus, the proposed RS LDPC codes are $(64{,}72)$-regular codes that have a codeword length of 18,432 and a dimension of 16,572 with a rate of 0.90 and a minimum distance of at least 66.

To encode the message bits to a codeword, we first make the parity check matrix into the full rank matrix.

Let H be the full rank matrix and defined as

$$\mathbf{H} = [\mathbf{A} \quad \mathbf{B}].$$

Then, we obtain the generator matrix $\mathbf{G}$ as

$$\mathbf{G}^{\mathbf{T}} = \begin{bmatrix} \mathbf{A}^{-1}\mathbf{B} \\ \mathbf{I} \end{bmatrix},$$

and

$$\mathbf{HG}^{\mathrm{T}} = \mathbf{0}.$$

Then, for the message $\mathbf{m}$, we can encode the message and obtain the codeword $\mathbf{c}$ by

$$\mathbf{c} = \mathbf{mG}.$$

If $\mathbf{c}$ is the valid codeword, the following equation holds.

$$\mathbf{Hc}^{\mathrm{T}} = \mathbf{H} \times \mathbf{G}^{\mathrm{T}}\mathbf{m}^{\mathrm{T}} = (\mathbf{HG}^{\mathbf{T}}) \times \mathbf{m}^{\mathrm{T}} = \mathbf{0} \times \mathbf{m}^{\mathrm{T}} = 0.$$

In this work, the rank of the parity check matrix $\mathbf{H}$ was 1,860, whose size was $1{,}860 \times 18{,}432$ had the size of $\mathbf{G}$ was $16{,}572 \times 18{,}432$. The size of the message is 16,572 bits and the length of the codeword is 18,432 bits. We encoded 272 messages and we obtained 18,432 binary sequences of length 272 bits.

<u>RS encoding of the index</u>

After we determined 18,432 indices, we protect them using RS codes. As will be mentioned later, since we perform index-based clustering during the decoding procedure, it is necessary to protect the index with strong error-correcting codes. RS codes are defined as $[n, k, n - k + 1]$ over $GF(q)$, the linear block code length is n with dimension k, and the minimum Hamming distance is $n - k + 1$. They are one of the maximum distance separable (MDS) codes, which achieve equality in the Singleton bound. Here, by adding 8 nt of RS redundancy to the 8 nt index, we obtain $(8{,}4{,}5)$ RS codes over $GF(2^4)$. Each symbol of RS codes is 4 nt (i.e., 2 nt), the codeword length is 8 symbols with the dimension 4, and the minimum Hamming distance is 5, which can correct up to 2 symbols and detect up to 4 symbols. By attaching the encoded index in the prefix of 18,432 oligo sequences.

Supplementary Notes 2

Decoding algorithm

Overview of the decoding algorithm

1. Randomly sample the forward and reverse reads.
2. Merge them with FLASH.
3. Save reads with lengths 150, 151, and 152 nt.
4. Detach the index and correct errors by RS decoding.
5. Gather reads with the same index (index-based clustering).
6. Discard reads that have a large edit distance compared to other reads in the same cluster (edit distance-based clustering).
7. Check conditions and align reads with MUSCLE.
8. Calculate LLR using Q-score values.
9. Perform LDPC decoding
10. For reads that are not corrected, perform LDPC re-decoding by changing ϵ.

Fig. S2-2 shows the overall decoding algorithm of the proposed method.

Merging, clustering, and alignment

For merging the forward and reverse reads, we used paired-read merger FLASH [5]. We used version FLASH-1.2.11 from http://ccb.jhu.edu/software/FLASH/. Table S1 shows the percentages of merged reads according to the length of merged reads using FLASH. Then, we chose reads with lengths 150, 151, and 152 nt that are used for the decoding, after the merging step. To perform index-based clustering, we detached the first 16 nt index from all chosen reads, corrected errors with RS decoding, and performed index and edit distance-based clustering. For the multiple sequence alignment (MSA) algorithm, we employ MUSCLE [6]. We used the version MUSCLE version 5.1 from https://github.com/rcedgar/muscle/releases/tag/v5.1. All merging, clustering, and alignment steps are implemented by Python.

Using Q-score values and LDPC re-decoding

In this work, we use LDPC soft decision decoder to retrieve the original data using sequenced reads. Unlike a hard decision decoder that operates decoding through definite values (e.g., 0 or 1 for binary codes), a soft decision decoder operates decoding by considering a range of possible values for each bit position. A soft decision decoder considers the reliability of each bit position and this gives additional information to the decoder for better estimation. This characteristic enables us to give a high input value for the reliable base or discard oligo sequences that are likely to be erroneous. In this work, to aid the decoder for better performance, we use Q-score information while computing LLR. Since it is known that asymmetric errors occur in DNA-based data storage as shown in Fig. S3 [8], the last base of the oligo sequence is more prone to errors compared to other positions. Thus, in the practical experiment, the last codeword is the bottleneck of the decoding performance. The last LDPC codeword is likely to

have more errors compared to other codewords and we use the Q-score value during the decoding of the last LDPC codeword to cope with this problem.

We first used Q-score values during the clustering step. For clusters of size one with shortened read, we cannot determine where deletion error occurred when only one read exists in the cluster. However, unless the deletion or substitution error occurs in the last base, the last base stays safe. Then, the next step is to figure out whether the error exists in the last base. We use the Q-score value of the last base to do this work. If the Q-score is higher than 30, we trust the last base and if not, we discard the oligo sequence.

Also, during the LLR calculation, we used the Q-score values. The LLR calculation considering the LDPC re-decoding scheme as follows:

$$\mathrm{LLR}^{\mathrm{i}}(k_0, k_1) = \ln \frac{P^{\mathrm{i}}\big((k_0, k_1, k_{\mathrm{e}}) \mid 0 \big)}{P^{\mathrm{i}}\big((k_0, k_1, k_{\mathrm{e}}) \mid 1 \big)} = \ln \frac{P^{\mathrm{i}}\big((k_0, k_1) \mid 0 \big)}{P^{\mathrm{i}}\big((k_0, k_1) \mid 1 \big)} = (k_0 - k_1) \ln \frac{1 - (\epsilon - i\Delta\epsilon)}{(\epsilon - i\Delta\epsilon)},$$

where k_0, k_1, and k_e denote counts of 0's, 1's, and erasures at the same position of reads in the same cluster and $k_0 + k_1 + k_e$ is the number of reads in the cluster and i denotes the number of iterations in the LDPC re-decoding. However, the erased bit does not affect the value of LLR. When counting the number of 0's and 1's in the last codeword, we checked the Q-score value. If the Q-score is lower than 20, we do not trust it and do not include it in the counting. Since the last LDPC codeword is the bottleneck of the experiment, we were able to improve the decoding performance by discarding unreliable bases using the Q-score.

After the first decoding of LDPC codes, we performed the re-decoding stage to maximize the decoding performance. According to the equation, we have to choose the value of the parameter ϵ. The initial value is $\epsilon_{init} = 0.02$, whose initial LLR value becomes

$$LLR_{init}(k_0, k_1, k_e) = (k_0 - k_1) \times 3.8918.$$

In the re-decoding step, we performed iterative decoding by changing ϵ values by subtracting ϵ by $\Delta\epsilon = 0.0005$ until the decoding succeeds or ϵ reaches the value 0.001. Since LDPC codes can determine whether the decoded codeword is correct or not by using the parity check matrix, we were able to perform the re-decoding stage only for decoded codewords with errors. Fig. S4 shows the brief flow chart of the decoding procedure. Also, the comparison of previous works and this work is shown in Table S2.

Supplementary Notes 3

DNA synthesis and sequencing

Synthesis

The DNA oligo pool consisting of 18,432 oligo sequences with 200 nt, including primers, was synthesized by Twist Bioscience.

- **Sequencing primers**

5’-GTTCAGAGTTCTACAGTCCGACGATC [152 nt] TGGAATTCTCGGGTGCCAAGG-3’

- **PCR primers (RP1)**

5’-AATGATACGGCGACCACCGAGATCTACACGTTCAGAGTTCTACAGTCCGA

- **PCR primers (RPI1)**

5'-CAAGCAGAAGACGGCATACGAGATCGTGATGTGACTGGAGTTCCTTGGCACCCGAGAATTCCA

Sequencing

For more accurate and reliable results, we perform three times of DNA sequencing for the same DNA oligo pool. To have the same sequencing environment as previous works [3] and [4], we follow exactly the same sequencing procedure as these works. We use Q5 Hot Start High-Fidelity 2X Master Mix with Illumina small RNA primers RP1 and RPI1, which are mentioned above, for PCR (2.5ul of each primer (10μM), 25ul Q5 Master Mix in a 50ul reaction). Thermocycling conditions for PCR: 97°C for 30 s, 10 cycles of 98°C for 10 s, 60°C for 30 s, 72°C for 30 s, 5 min of extension at 72°C. The library is purified 1:1 and cleaned up with Agencourt AMPure XP and eluted in 20ul water. The sequencing is performed using Illumina Miseq Reagent v3 kit (600 cycles) with 150 pair-end reads in both forward and reverse directions.

Supplementary Notes 4

Comparison of the information density, writing cost and reading cost to previous works

Information density

The information density is defined as "How many information bits are stored in one base after the encoding?". In this work, the size of the payload is $16{,}572 \times 272$ bits. These binary sequences are converted to $18{,}432 \times 152$ nt after attaching LDPC parity, RS parity, and index. Let R_{proposed} be the information density of the proposed encoding algorithm and it is defined as

$$R_{\text{proposed}} = \frac{16{,}572 \times 272 \text{ bits}}{18{,}432 \times 152 \text{ nt}} = 1.61 \text{ bits/nt}.$$

In this work, we compare the information density with the experiment conducted in [4]. They apply the encoding method of DNA Fountain codes and measure the decoding performance using Erlich's decoder [3] and Jeong's decoder [4]. They use LT codes as inter-oligo codes and RS codes as intra-oligo codes. They partitioned 513.6 KB of the image into 16,050 binary sequences of length 256 bits. Then, they encoded binary sequences into 18,432 DNA sequences of length 152 nt. Let $R_{[4]}$ be the information density of the DNA Fountain encoding algorithm applied in [4]. Then, $R_{[4]}$ is defined as

$$R_{[4]} = \frac{16{,}050 \times 256 \text{ bits}}{18{,}000 \times 152 \text{ nt}} = 1.50 \text{ bits/nt}.$$

Writing cost

As mentioned in the paper, the writing cost is defined by the number of synthesized bases divided by the number of information bits. This shows that the writing cost is the inverse of the information density. In this work, 18432×152 nt of DNA bases are used to store 16572×272 bits of data. Let WC be the writing cost of this work. Then it can be written as

$$\text{WC} = \frac{18432 \times 152}{16572 \times 272} = \frac{1}{R_{\text{proposed}}} = 0.62 \text{ bases/bit}.$$

In the same way, let $\text{WC}_{[4]}$ be the writing cost of the work [4]. Then $\text{WC}_{[4]}$ can be also written as

$$\mathrm{WC}_{[4]} = \frac{18000 \times 152}{16050 \times 256} = \frac{1}{R_{[4]}} = 0.67 \text{ bases/bit.}$$

Also, the writing cost reduction compared to [4] can be calculated as follows:

$$\frac{(\mathrm{WC}_{[4]} - \mathrm{WC})}{\mathrm{WC}_{[4]}} = \frac{(0.67 - 0.62)}{0.67} \approx 0.0746$$

Compared to the previous work [3], this work achieved 7.46% of writing cost reduction with the same synthesis provider (Twist Bioscience)

Reading cost

The reading cost is defined by the number of sequenced bases divided by the number of information bits. In [4], they measure the decoding performance using Erlich's [3] and Jeong's [4] decoders for the same input data file. Let RC_1, RC_2, and RC_3 be the reading costs of this work in Exp #1, #2, and #3. Then, it can be written as

$$\mathrm{RC}_1 = \frac{72500 \times 152}{16572 \times 272} = 2.44 \text{ bases/bit,}$$

$$\mathrm{RC}_2 = \frac{71500 \times 152}{16572 \times 272} = 2.41 \text{ bases/bit,}$$

$$\mathrm{RC}_3 = \frac{73500 \times 152}{16572 \times 272} = 2.48 \text{ bases/bit.}$$

In [7], they have writing and reading costs of 0.67 bases/bit and 3.82 bases/bit for LDPC redundancy of 10% to restore 224 KB of data.

Let $\mathrm{RC}_{[3]}$ and $\mathrm{RC}_{[4]}$ be the reading costs of using Erlich's [3] and Jeong's [4] decoders. They are defined as follows:

$$\mathrm{RC}_{[3]} = \frac{90000 \times 152}{16050 \times 256} = 3.33 \text{ bases/bit,}$$

$$\mathrm{RC}_{[4]} = \frac{82000 \times 152}{16050 \times 256} = 3.03 \text{ bases/bit.}$$

Compared to previous works using Erlich's and Jeong's decoders, this work achieved 26.57% and 19.41% of reading cost reduction using the same sequencing kit (Illumina Miseq v3 kit) in the same sequencing environment.

Also, the average writing cost reduction compared to Erlich's and Jeong's decoder is as follows:

$$\frac{(\mathrm{RC}_{[3]} - \mathrm{RC})}{\mathrm{RC}_{[3]}} = \frac{(3.33 - 2.44)}{3.33} \approx 0.2657,$$

$$\frac{(\mathrm{RC}_{[4]} - \mathrm{RC})}{\mathrm{RC}_{[4]}} = \frac{(3.03 - 2.44)}{3.03} \approx 0.1941.$$

Supplementary Notes 5

Additional discussion

The coverage of the DNA-based data storage experiment

In DNA-based data storage, the random sampling and merging steps can be modeled as Poisson distribution.

This coverage distribution can be modeled as follows.

$$P(X = k) = \frac{\lambda^k e^{-\lambda}}{k!},$$

where k is a coverage and λ is the mean of the Poisson distribution. Then, λ is defined as follows:

$$\lambda = \frac{\text{Number of randomly sampled oligo sequences}}{\text{Number of the encoded oligo sequences}}.$$

Let λ_i for $i = 1,2,3$ be the mean of the ideal Poisson distribution $P_i(X = k)$ for Exp \#1, \#2, and \#3, respectively. For the random sampling number at which all 200 trials of the decoding succeed, $\lambda_1 = 72500/18432$, $\lambda_2 = 71500/18432$, and $\lambda_3 = 73500/18432$. Then, the ideal Poisson distribution can be plotted as in Fig. S5.

Supplementary Tables

Supplementary Table S1. The percentages of merged reads according to the length of merged reads

Merged read lengths	Pool #1	Pool #2	Pool #3
150 nt	0.24%	0.24%	0.27%
151 nt	1.07%	1.09%	1.18%
152 nt	93.01%	93.36%	92.77%
153 nt	0.77%	0.78%	0.77%
154 nt	0.01%	0.01%	0.01%
≥ 155nt	0.09%	0.07%	0.09%
Used reads	94.32%	94.69%	94.22%
Total	95.19%	95.54%	95.08%

The percentages of merged reads are over 95% on average. Since the percentages of the shortened reads are higher than reads with lengths over 152 nt, the proposed scheme uses additional shortened reads only. Thus, from merged reads, the proposed scheme uses reads with length 150, 151, and 152 nt, which are over 94% of sequenced reads. In other words, the proposed scheme uses 1.3% of additional reads compared to the scheme using only the correct length reads.

Supplementary Table S2. Comparison between previous works and the proposed decoder.

Methods	Writing cost (bases/bit)	Information density (bits/nt)	Coverage	Sequencing	Data size
[3]	0.64	1.57	x 10.5	Illumina	2.14 MB
Jeong's [4]	0.67	1.50	x 5	Illumina	513 KB
Erlich's [4]	0.67	1.50	x 4.56	Illumina	513 KB
[7]	0.67	1.49	x 5.69	Illumina	224 KB
[8]	0.91	1.10	x 5	Illumina	200 MB
[9]	1.2	0.83	x 3000	Illumina	650 KB
[10]	3.45	0.29	x 51	Illumina	739 KB
[11]	0.86	1.16	x 372	Illumina	84 KB
**Our work**	**0.62**	**1.61**	**x 3.93**	**Illumina**	**549 KB**

The comparison of writing cost, information density, coverage, sequencing technique, and data size between previous works [3], [4], [7], [8], [9], [10], [11], and the proposed method. Our work achieves the best result in terms of the writing cost and coverage.

Supplementary Figures

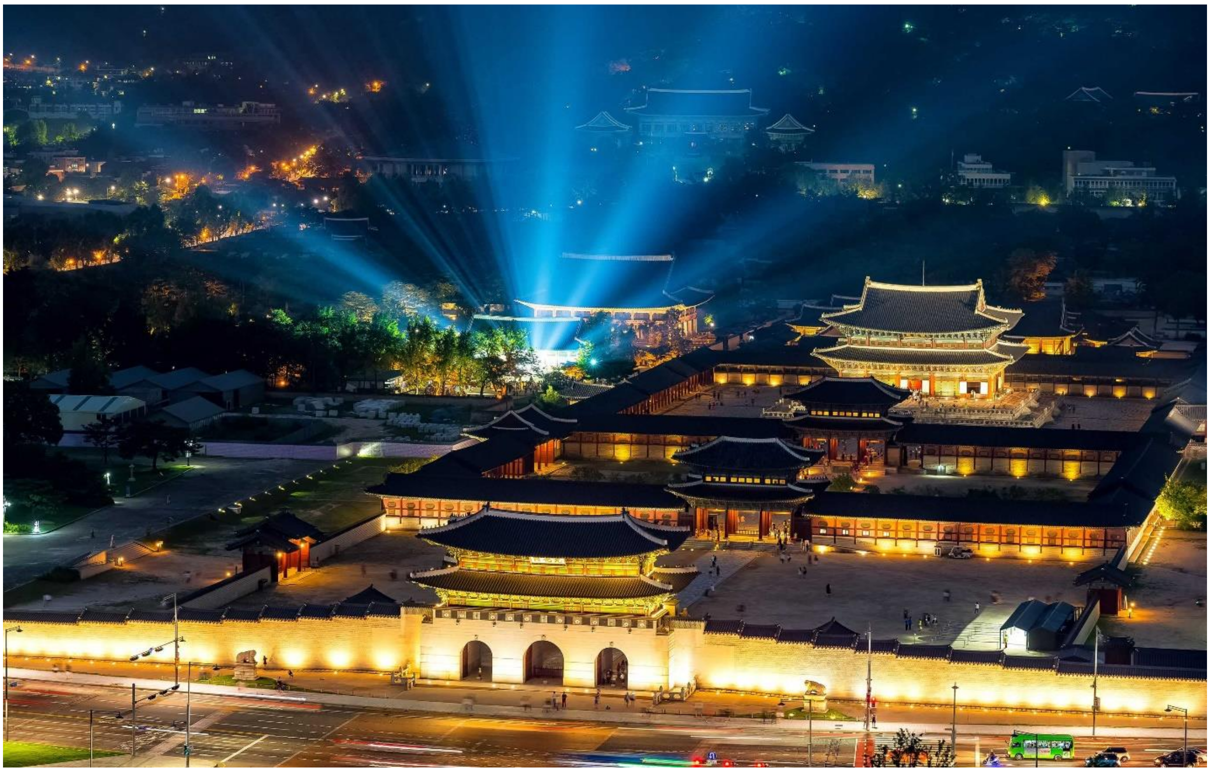

Fig. S1: The image of 584.83 KB of Gyeongbokgung Palace in Seoul, Republic of Korea. This image is from https://en.wikipedia.org/wiki/Gyeongbokgung.

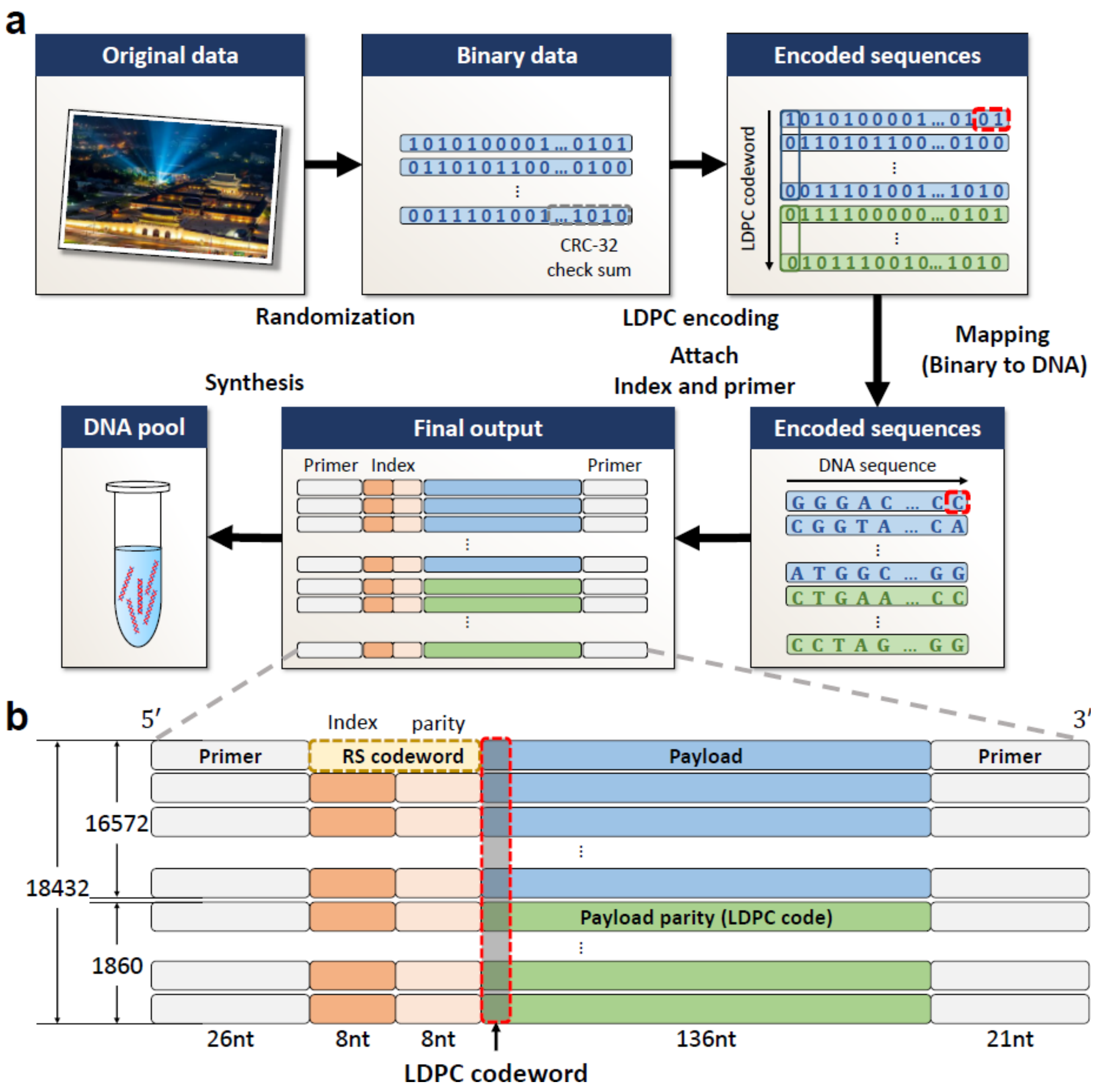

Fig. S2-1: Overall encoding procedure of the proposed sequence and the encoding output. **(a)** The original data is first converted to a binary sequence and partitioned into short sequences. Then, LDPC codes are employed in inter-oligo direction to generate parity sequences and convert each binary sequence to DNA sequences. For each oligo sequence, the protected index is added. Finally, primers are added and the DNA oligo sequences are synthesized. **(b)** The encoding output of the original data. 16,572 oligo sequences are from the data payload and 1860 oligo sequences are generated by LDPC encoding. Each sequence consists of 26 nt primer, 8 nt index, 8 nt RS parities for the index, 136 nt data payload or LDPC parity, and 21 nt primer. Finally, 18,432 oligo sequences with length 199 nt are generated after the encoding procedure.

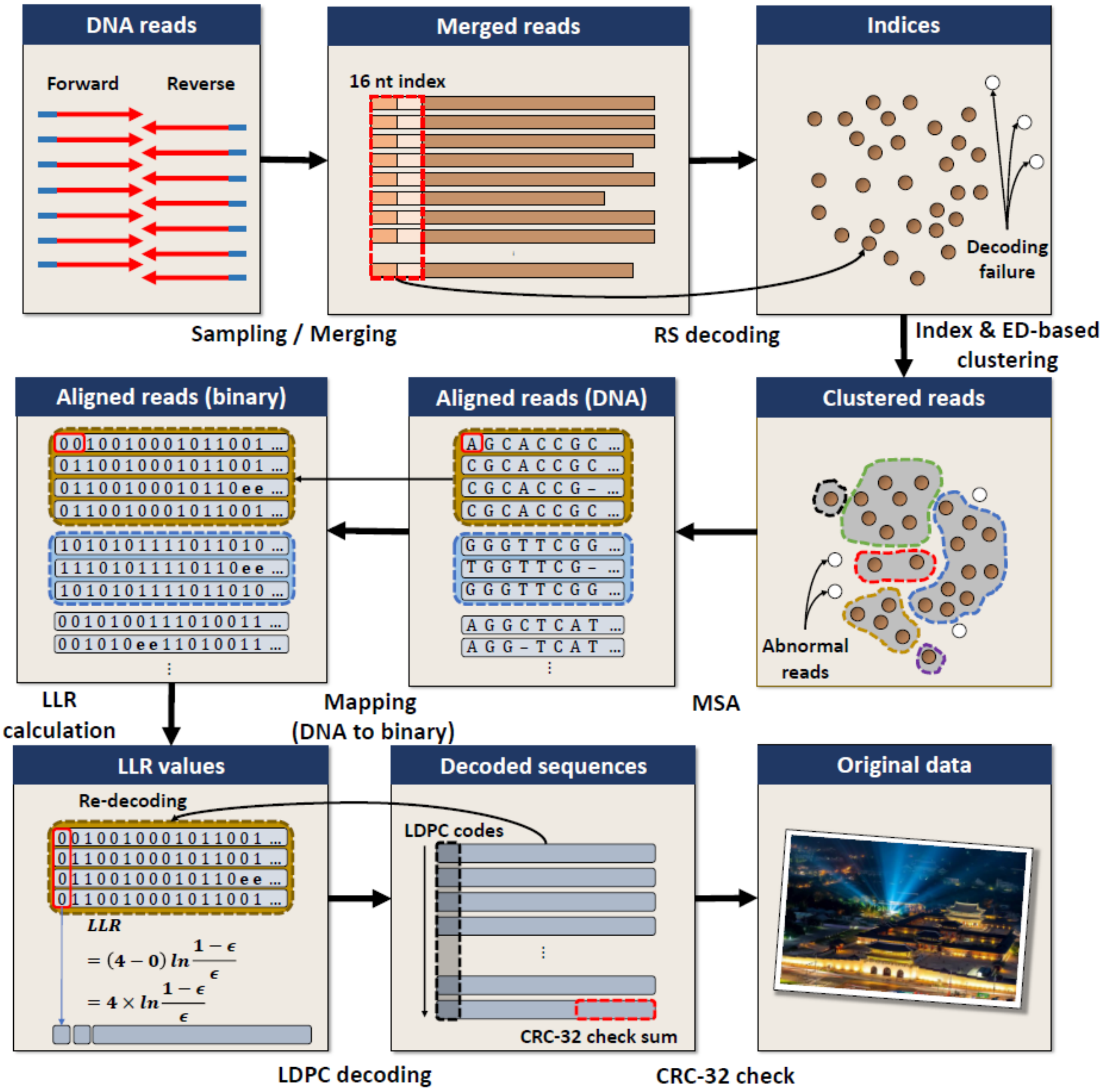

Fig. S2-2: Overall decoding algorithm of the proposed scheme. From merged reads, reads with lengths 150, 151, and 152 nt are selected for decoding. The index of all selected reads is first decoded and reads with the same index are grouped into the same cluster. Then, for all reads in each cluster, the ED between each pair of reads is calculated and reads which have large EDs are discarded. For clusters with VL reads, multiple sequence alignment is applied to convert the reads to binary sequences. Next, LLR values for each aligned read are calculated and become inputs at the decoding of LDPC codes. From the decoded output, the original data after checking the CRC-32 are retrieved.

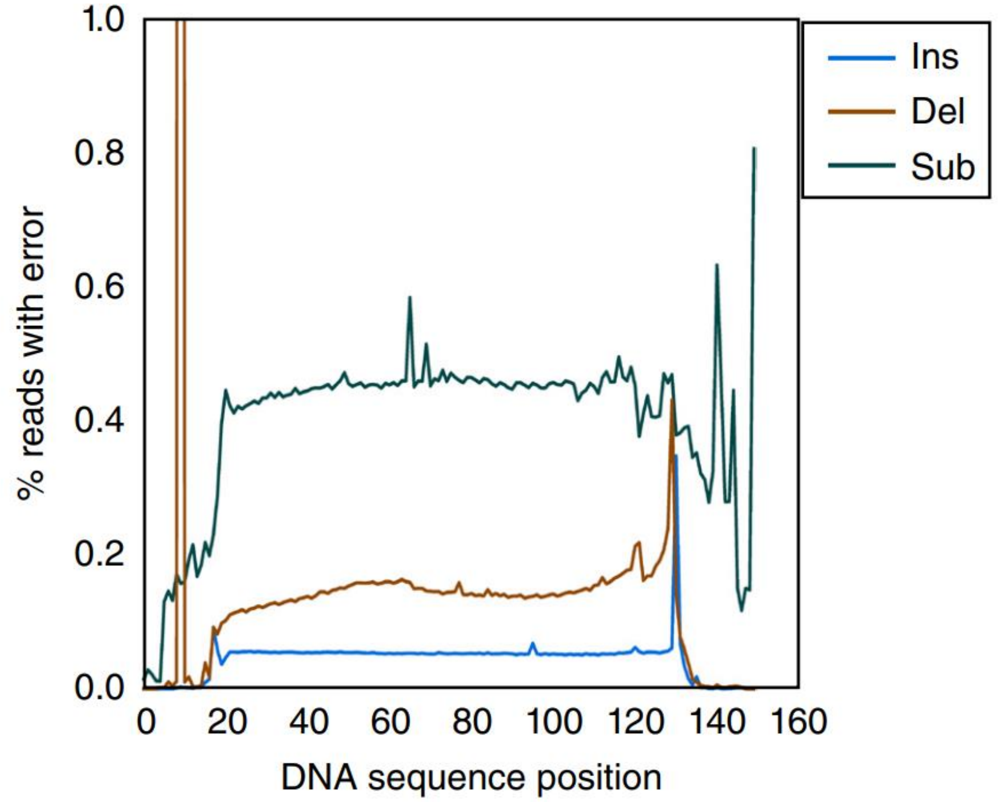

Fig. S3: The figure from Fig. 3(b) in [8]. Per-position average read error profile for the first 150 positions in DNA sequences. DNA-based data storage has asymmetric errors and the error rate of the last base is higher than the error rate in other positions.

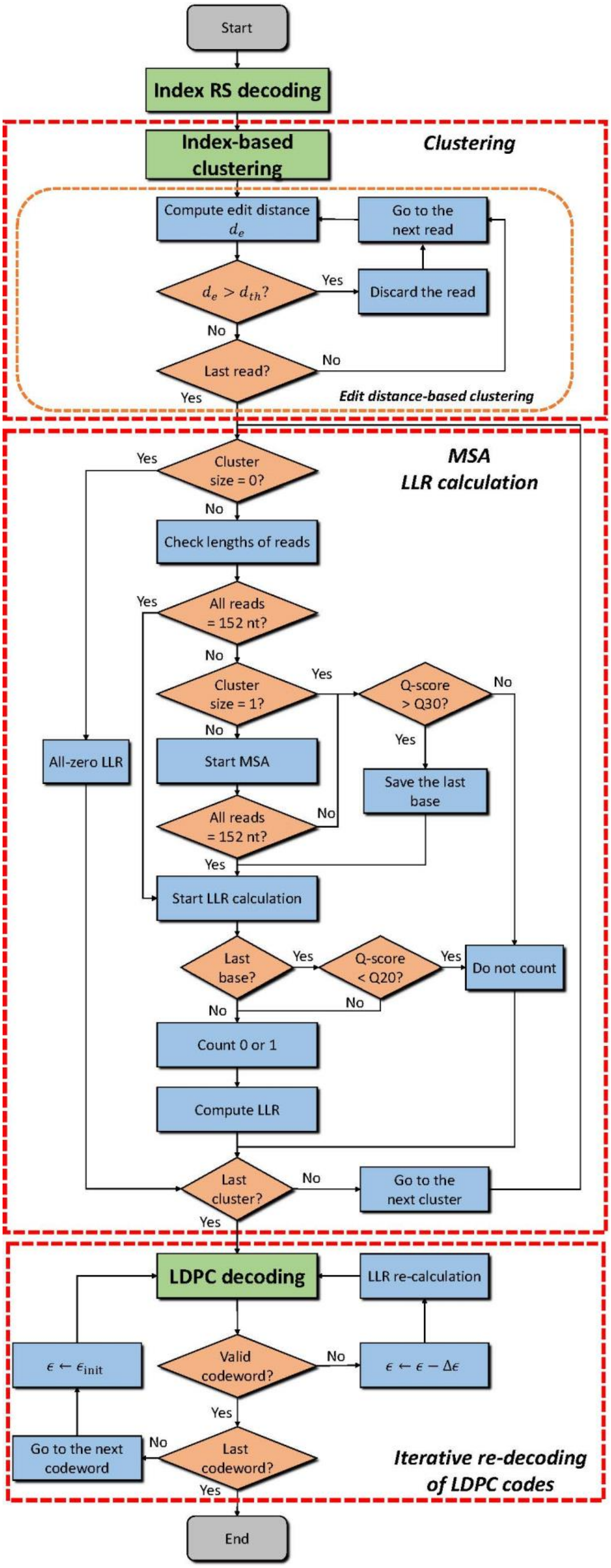

Fig. S4: A brief flow chart of the decoding procedure. The top red dotted box shows the clustering step, the middle red dotted box shows the MSA and LLR calculation steps and the bottom red dotted box shows the iterative re-decoding of LDPC codes. The orange dotted box shows the edit-distance based clustering algorithm

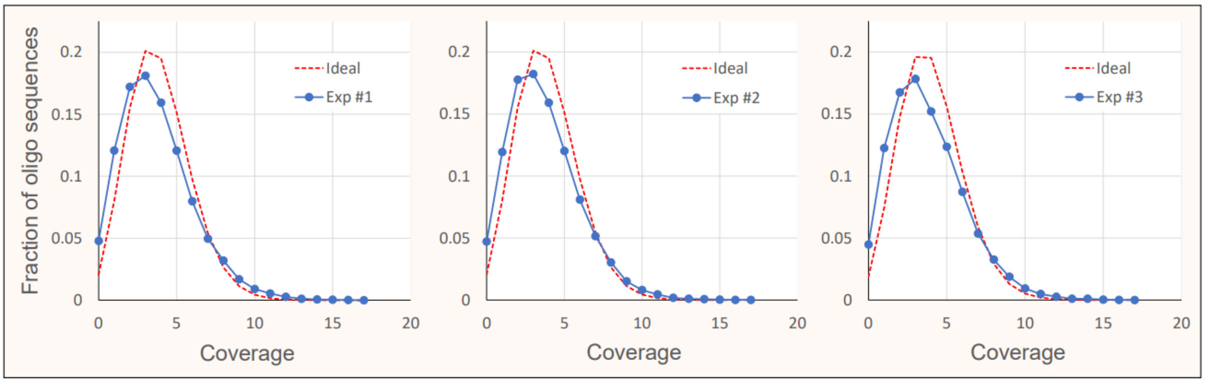

Fig. S5: The coverage histogram for the ideal Poisson sampling and the three DNA-based data storage experiments. The mean coverages for the three experiments are 3.93, 3.88, and 3.99, respectively.

References

[1] Richardson, T. J. & Urbanke, R. (2008) *Modern Coding Theory*. Cambridge Univ. Press, Cambridge, U.K.

[2] Djurdjevic, I., Xu, J., Abdel-Ghaffar, K., & Lin, S. (2003) A class of low-density parity-check codes constructed based on Reed–Solomon codes with two information symbols. *IEEE Commun. Lett.*, **7**, 317–319.

[3] Erlich, Y. & Zielinski, D. (2017) DNA fountain enables a robust and efficient storage architecture. *Sci.*, **355**, 950–954.

[4] Jeong, J. et al. (2021) Cooperative sequence clustering and decoding for DNA storage system with fountain codes. *Bioinformatics*, **37**, 3136–3143.

[5] Magoč,T. & Salzberg, S. L. (2011) FLASH: Fast length adjustment of short reads to improve genome assemblies. *Bioinformatics*, **27**, 2957–2963.

[6] Edgar, R. C. (2004) MUSCLE: Multiple sequence alignment with high accuracy and high throughput. *Nucleic Acids Res.*, **32**, 1792–1797.

[7] Chandak, S. et al. (2019) Improved read/write cost tradeoff in DNA-based data storage using LDPC codes. In: *2019 57th Annual Allerton Conference on Communication, Control, and Computing (Allerton)*.

[8] Organick, L. et al. (2018) Random access in large-scale DNA data storage. *Nat. Biotechnol.*, **36**, 242–248.

[9] Church, G. M., Gao, Y., and Kosuri, (2012) S. Next-generation digital information storage in DNA. *Sci.*, **337**, 1628–1628.

[10] Goldman, N. et al. (2013) Towards practical, high-capacity, lowmaintenance information storage in synthesized DNA. *Nat.*, **494**, 77–80

[11] Grass, R., Heckel, R., Puddu, M., Paunescu, D. and Stark, W. J. (2015) Robust chemical preservation of digital information on DNA in silica with error-correcting codes. *Angewandte Chemie Int.*, **54**, 2552–2555.
